# Bacterial profile and antibiotic susceptibility pattern of uropathogens causing urinary tract infection in the eastern part of Northern India

**DOI:** 10.3389/fmicb.2022.965053

**Published:** 2022-08-09

**Authors:** Kanika Bhargava, Gopal Nath, Amit Bhargava, Ritu Kumari, G. K. Aseri, Neelam Jain

**Affiliations:** ^1^Amity Institute of Microbial Technology, Amity University Rajasthan, Jaipur, India; ^2^Department of Microbiology, Institute of Medical Sciences (IMS), Banaras Hindu University, Varanasi, India; ^3^Department of Medicine, Hayes Memorial Hospital, Sam Higginbottom University of Agriculture, Technology and Sciences (SHUATS), Allahabad, India; ^4^Amity Institute of Biotechnology, Amity University Rajasthan, Jaipur, India

**Keywords:** antibiotic susceptibility (AS), antimicrobial resistance (AMR), multidrug resistance (MDR), urinary tract infections, uropathogens, India

## Abstract

Urinary tract infection (UTI) is a common infectious disease that affects men and women. It is a significant health concern due to multidrug-resistant (MDR) organisms. Therefore, it is necessary to have a current understanding of the antibiotic susceptibility (AS) pattern of uropathogens to manage UTI effectively. Since the bacterial pathogen causing UTI and its AS vary with time and place, the prevailing AS pattern of the causative agents are essential for empirical antibiotic therapy. This study aims to determine the prevalence and AS of uropathogens isolated from UTI patients in the eastern part of Northern India. The study was carried out between November 2018 and December 2019. Clean catch midstream urine samples were collected and processed using standard guidelines for microbiological procedures. Positive microbiological cultures were found in 333 of the 427 patients, where 287 were gram-negative bacteria (GNB), and 46 were gram-positive bacteria (GPB). Females had a higher prevalence of UTI (60.7%) than males (39.3%) (*p* = 0.00024). The most susceptible age group in females was 18–50 years as compared to males, whereas at the age of 51–80 years and >80 years males were more susceptible than females (*p* = 0.053). The most prevalent pathogen identified were *Escherichia coli* (55.0%), followed by *Proteus* sp. (6.9%), *Klebsiella pneumoniae* (6.6%), *Pseudomonas aeruginosa* (6.3%), of which 96.0% were MDR bacteria. The susceptibility pattern of our study also revealed that amikacin, gentamycin and imipenem were the most effective drugs against GNB. In contrast, nitrofurantoin, vancomycin, and chloramphenicol were the most effective drugs against GPB. According tothe findings, MDR pathogens are very much prevalent. Since UTI is one of the most frequent bacterial diseases, proper management necessitates extensive investigation and implementation of antibiotic policy based on AS patterns for a particular region.

## Introduction

Urinary tract infections (UTIs) are inflammatory disorders caused by microorganisms that have proliferated abnormally in the urinary system (Malik et al., 2021). UTIs are known to induce short-term morbidities such as fever, dysuria, lower abdominal pain, and may result in permanent kidney scarring (Leung et al., 2019). UTIs are either community-acquired or hospital-acquired (HA). Infection of the urinary system originates in individuals either in the community (within 48 h of admission) or a hospital setting (Revelas, 2012). HA-UTI emerges 48 h after hospitalization and is not incubating at the time of admission or within 3 days of discharge (Iacovelli et al., 2014; Motbainor et al., 2020). UTIs can be asymptomatic or symptomatic, imposing a strain on public health care and lowering the quality of life (Olowe et al., 2015).

Urinary tract infection is more common in women than in men because of the anatomical proximity of the urethra to gut opening (Fazly Bazzaz et al., 2021). The most prevalent bacteria causing UTI is *Escherichia coli*, followed by *Klebsiella pneumoniae*, *Staphylococcus* sp., *Proteus* sp., *Pseudomonas aeruginosa, Enterococcus* sp., and *Enterobacter* sp. with variations in their sequence of prevalence (Ahmed et al., 2019; Patel et al., 2019; Mukherjee et al., 2020). Approximately 150 million UTI cases per year are diagnosed globally, resulting in at least $6 billion in healthcare costs (Kucheria et al., 2005; Flores-Mireles et al., 2015). Susceptibility data from local microbiological facilities assist in the empirical selection of antibiotics for UTI treatment; however, these data are confined to complicated UTIs because uncomplicated UTI specimens are rarely sent to laboratories (Prakash and Saxena, 2013). Therefore, UTIs are currently treated empirically, particularly in rural and small-town settings where the facility of urine culture is unavailable, resulting in antibiotic misuse (Al-Zahrani et al., 2019). The increasing incidence of drug resistance among uropathogens is a significant public health concern, necessitating constant antibiotic susceptibility (AS) screening for organisms causing UTI (Kot, 2019). In addition, antimicrobial sensitivity for UTI-causing bacteria varies with time and location. Therefore, screening for susceptibility in each location is critical for producing up-to-date epidemiological data (Ahmed et al., 2019; Daoud et al., 2020). Unfortunately, the resistance profile of community-acquired uropathogens in diverse geographical regions of India has not been adequately explored (Sood and Gupta, 2012; Mohapatra et al., 2022). Since UTIs are frequently treated empirically in regions where microbiological facilities are either unavailable or prohibitively expensive for the majority of the Indian population, treatment is based on the anticipated pathogens with their AS pattern of that geographic area. We chose to conduct this study because we were unaware of the bacterial composition and AS pattern of uropathogens causing UTI in Prayagraj (Uttar Pradesh), India, which is situated in the eastern region of North India.

## Materials and methods

### Study area and population

A cross-sectional study was conducted at Hayes Memorial Mission Hospital in Prayagraj, Uttar Pradesh, between November 2018 and December 2019 to investigate the prevalence and AS profile of uropathogens among patients presenting with UTI. Sample size was calculated by Kish (1965) formula, *n* = z^2^p(1-p)/d^2^, where z = Z score for 95% confidence interval = 1.96; p = prevalence (22.8%) and d = acceptable error (5%). The formula also included 1.5 times the design effect and a 5% non-response rate. A total of 427 samples were acquired based on a subjective symptom-based questionnaire [data not shown], of which 333 were later verified microbiologically as positive UTI cases.

### Exclusion criteria

Patients under the age of five, those with polymicrobial infections involving more than two bacterial species, patients with *Candida* sp. as the sole pathogen or with bacteria, pregnant females with asymptomatic bacteriuria, and those who had previously been on antibiotic therapy were all excluded from the study.

### Sample collection and processing

Each patient’s clean-catch midstream urine was collected in a sterile screw-capped universal container. All patients were instructed on collecting samples aseptically to avoid contamination. A urine sample is medical waste material voluntarily given by patients visiting OPD, without invasive sample collection procedures. However, patients’ oral and/or written consent was also collected before specimen collection and the study was approved by institutional committee. A sterile calibrated loopful of urine sample was plated on sheep blood agar (SBA) and MacConkey agar (MA) to isolate bacterial uropathogens and incubated at 37°C for 24 h.

### Identification and biochemical characterization of pathogens

Bacterial isolates were identified based on their standard microbiological techniques, i.e., culture and biochemical characteristics. All the bacteria isolated from the sample were identified using catalase test (3% v/v H_2_O_2_), coagulase test (0.85% v/v of normal saline), bile esculin test, oxidase test, indole test with H_2_S production (sulphide indole motility medium), citrate utilization test (Simmon’s citrate medium), urease test (Christensen’s urea agar), triple sugar iron agar test and fermentation using sugars (Glucose, Lactose, Sucrose, and Mannitol). Isolates identified were preserved at room temperature of 25°C in peptone soft agar that was wax sealed with a cork and sub-cultured for further processing.

### Antimicrobial susceptibility

The antimicrobial susceptibility test was performed on Mueller–Hinton agar (HiMedia Laboratories, Mumbai, India) using the Kirby–Bauer disk diffusion method and interpreted according to Clinical Laboratory Standards Institute (CLSI) guidelines (Table 1). Extended-spectrum beta-lactamase (ESBL) producing strains were confirmed by utilizing a double-disk synergy test with cephalosporin and cephalosporin/clavulanate combination disks (ceftazidime and ceftazidime-clavulanic acid) for *E. coli* and *K. pneumoniae*. Standard strains of *E. coli* (ATCC 25922), *Staphylococcus aureus* (ATCC 25923), and *Pseudomonas aeruginosa* (ATCC 27853) were used in this study as quality control.

**TABLE 1:** Antibiotics used against different isolated uropathogens.

Antibiotics groups		Antibiotic
B eta-lactams	Penicillin	Ampicillin
		Penicillin
		Piperacillin
	Cephalosporin	Cefepime
		Ceftazidime
		Cefoxitin
		Ceftriaxone
	Carbapenem	Aztreonam
		Meropenem
		Imipenem
Beta-lactamase inhibitors		Piperacillin-Tazobactam
		Ceftazidime-Clavulanic
		Amoxicillin-clavulanate (Amoxyclav)
Fluoroquinolones		Norfloxacin
		Ciprofloxacin
Aminoglycosides		Gentamycin
		Amikacin
		Gentamycin (120)
		Tobramycin
		Netilmicin
Glycopeptide		Vancomycin
		Teicoplanin
Tetracycline		Doxycycline
		Tetracycline
Others		Clindamycin
		Chloramphenicol
		Linezolid
		Erythromycin
		Nitrofurantoin
		Co-trimoxazole

### Statistical analysis

The data were analyzed using descriptive statistics for UTI prevalence, uropathogen frequency, AS profile, Chi-square test where applicable. All statistical tests were performed using SPSS software version 23 and Microsoft Excel 2016 (Microsoft Corporation, Redmond, Was, United States).

## Results

**FIGURE 1:**
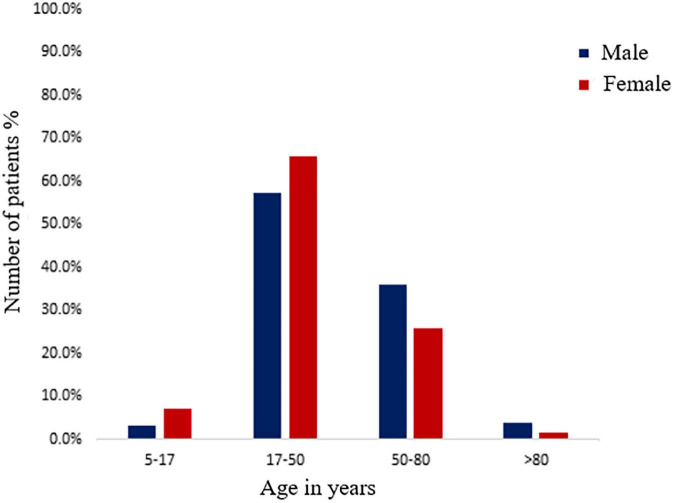
Distribution of male and female positive urinary tract infection (UTI) patients among different age groups.

Of the whole study group of 427, 333 (77.9%) were excreting a significant number of bacteria in their urine. Our study shows that 39.3% (131/333) males and 60.7% (202/333) of females were suffering from UTIs (χ^2^ = 13.495; degree of freedom = 1; *p* = 0.00024). The prevalence of UTI in females was significantly higher than the males (*p* = 0.00024). The most susceptible age group for UTI was 18–50 years, followed by 51–80, 5–17, and >80 years (Figure 1). However, at the age of 51–80 years (35.9%) and >80 years (3.8%), males had a higher prevalence of UTI than females (25.7 and 1.5%), but in the childhood and adolescent age, group females were more susceptible. The chi-square test showed a significant association between age group and gender (χ^2^ = 7.69; degree of freedom = 3; *p* = 0.053). A total of 333 bacterial uropathogens comprising of 287 (67.2%) gram-negative and 46 (10.8%) gram-positive bacteria were isolated from positive urine samples. There were nine different uropathogens isolated, six of which were gram-negative bacteria, and three were gram-positive bacteria. *E. coli* was the most predominant gram-negative bacteria, accounting for 54.95% (183/333) of all isolates, followed by *Proteus sp*. 6.9%, *K. pneumoniae* 6.6%, *P. aeruginosa* 6.3%, *Citrobacter* sp. 6.3%, *S. aureus* 6.0%, *Enterococcus* sp. 5.4%, *E. cloacae* 5.1% and *S. epidermidis* 2.4%. Also, gender (*p* = 0.620) had no significant association with the types of bacterial pathogens isolated (Table 2). The highest number of *E. coli* was found in the age group of 18–50 years (58.17%, 121/208) followed by 51–80 years (51.5%, 51/99), 5–17 years (50%, 9/18), >80 years (25%, 2/8). The second most prevalent organism among the age group of 18–50 years was *Proteus* sp., 7.2% (15/208), followed by *S. aureus*, 6.7% (14/208); *P. aeruginosa*, 5.8% (12/208), *Citrobacter* sp., 5.8% (12/208); *K. pneumoniae*, 5.3% (11/208); *E. cloacae*, 4.3% (9/208); *Enterococcus* sp., 3.8% (8/208); *S. epidermidis*, 2.9% (6/208). For 51–80 years, the second most prevalent organism is *K. pneumoniae*, 8.1% (8/99), *Proteus* sp., 8.1% (8/99) followed by *E. cloacae*, 7.1% (7/99); *Enterococcus* sp., 6.1% (6/99), *Citrobacter* sp., 6.1% (6/99), *S. aureus*, 6.1% (6/99); *P. aeruginosa*, 5.1% (5/99), and *S. epidermidis*, 2.0% (2/99). Also, for 5–17 years, the second most prevalent organism is *P. aeruginosa* 16.7% (3/18), *K. pneumonia*e, 16.7% (3/18); followed by *Enterococcus sp*., 11.1% (2/18) and *E. cloacae*, 5.6% (1/18). A significant association was found among the age group (*p* = 0.039) with respect to bacterial isolate (Table 3).

**TABLE 2:** Distribution of bacteria among gender in the study population.

Bacterial pathogens	Male (*n* = 131)	Female (*n* = 202)	Pearson Chi-square	*P*-value
*Escherichia coli*	51.9%	56.9%	6.244; df = 8	0.620
*Proteus* sp.	9.16%	5.44%		
*Klebsiella pneumoniae*	3.8%	8.41%		
*Pseudomonas aeruginosa*	6.84%	5.94%		
*Citrobacter* sp.	6.84%	5.94%		
*Staphylococcus aureus*	6.1%	5.94%		
*Enterococcus* sp.	6.1%	4.95%		
*Enterobacter cloaca*	6.84%	3.96%		
*Staphylococcus epidermidis*	2.29%	2.47%		

n, number; df, degree of freedom.

**TABLE 3:** Distribution of bacteria among the age groups of the study population.

Bacterial pathogens	5–17 years (*n* = 18)	18–50 years (*n* = 208)	51–80 years (*n* = 99)	>80 years (*n* = 8)	Pearson Chi-square	*P*-value
*Escherichia coli*	50%	58.17%	51.5%	5%	37.47; df = 24	0.039*
*Proteus* sp.	0%	7.2%	8.08%	0%		
*Klebsiella pneumoniae*	16.7%	5.28%	8.08%	0%		
*Pseudomonas aeruginosa*	16.7%	5.76%	5.05%	12.5%		
*Citrobacter* sp.	0%	5.76%	6.06%	37.5%		
*Staphylococcus aureus*	0%	6.7%	6.06%	0%		
*Enterococcus* sp.	11.1%	3.84%	6.06%	25%		
*Enterobacter cloacae*	5.55%	4.32%	7.07%	%		
*Staphylococcus epidermidis*	0%	2.88%	2.02%	0%		

n, number; df, degree of freedom; *Significant.

Antibiotic susceptibility testing revealed that 96.0% (320/333) of the pathogenic bacteria isolated from urine samples were MDR organisms. It was found that 40.4% (74/183) of *E. coli* and none of the *K. pneumoniae* were ESBL producing organisms. Table 4 demonstrates that aminoglycoside antibiotics such as amikacin and gentamycin were the most effective drugs. Amikacin was effective against 77.0% of *E. coli*, 73.9% of *Proteus* sp., 81.8% of *K. pneumoniae*, 52.9% of *E. cloacae*, 90.5% of *Citrobacter* sp., and 76.2% of *P. aeruginosa*. Gentamycin demonstrated an almost similar level of efficacy with susceptibility rates of 49.7% for *E. coli*, 56.5% for *Proteus* sp., 86.4% for *K. pneumoniae*, 52.9% for *E. cloacae*, 81.0% for *Citrobacter* sp., and 81.0% for *P. aeruginosa*. Tobramycin was tested only against *P. aeruginosa* isolates and was effective against 71.4% of them. With the exception of piperacillin-tazobactam and ceftriaxone, the isolates were relatively resistant to the β-lactam group of antibiotics, penicillin, and cephalosporins. Piperacillin-tazobactam inhibited 52.2% of *Proteus* sp., 71.4% of *Citrobacter* sp. and 71.4% of *P. aeruginosa*, as shown in Table 4, whereas, ceftriaxone was able to inhibit 42.9% of *Citrobacter sp*. isolates. The two carbapenem antibiotics also performed poorly, with meropenem showing efficacy against 52.4% of *Citrobacter* sp. but less than 50% of the other gram-negative isolates. However, imipenem outperformed meropenem by inhibiting 57.4% *E. coli*, 72.7% *K. pneumoniae*, 57.1% *Citrobacter* sp. and 90.5% *P. aeruginosa.* Nitrofurantoin, a nitrofuran antibiotic, was effective against a few bacteria, inhibiting 49.7% of *E. coli* and 61.9% of *Citrobacter* sp. Nitrofurantoin demonstrated poor susceptibility rates of 13.6 and 17.6% against *K. pneumoniae*, and *E. cloacae*, respectively (Table 4).

**TABLE 4:** Susceptibility of different antibiotics against isolated gram-negative and gram-positive uropathogens.

Antibiotics	*E. coli* (183)	*Proteus* sp. (23)	*K. pneumoniae* (22)	*E. cloacae* (17)	*Citrobacter* sp. (21)	*P. aeruginosa* (21)	*Enterococcus* sp. (18)	*S. aureus* (20)	*S. epidermidis* (8)

	Gram-negative uropathogens						Gram-positive uropathogens		
Amikacin	77.0%	73.9%	81.8%	52.9%	90.5%	76.2%	NT	NT	NT
Gentamycin	49.7%	56.5%	86.4%	52.9%	81.0%	81.0%	NT	NT	NT
Tobramycin	NT	NT	NT	NT	NT	71.4%	NT	NT	NT
Ampicillin	1.1%	8.7%	0.0	0.0	23.8%	NT	66.7%	NT	NT
Amoxy-clav	12.6%	0.0	18.2%	5.9%	4.8%	NT	NT	NT	NT
Piperacillin	NT	NT	NT	NT	NT	23.8%	NT	NT	NT
Piperacillin-tazobactum	37.7%	52.2%	45.5%	23.5%	71.4%	71.4%	NT	NT	NT
Ceftazidime	12.0%	26.1%	54.5%	17.6%	33.3%	23.8%	NT	NT	NT
Cefepime	6.6%	8.7%	22.7%	23.5%	9.5%	38.1%	NT	NT	NT
Ceftriaxone	16.9%	26.1%	40.9%	35.3%	42.9%	NT	NT	NT	NT
Co-trimoxazole	39.8%	34.7%	86.4%	35.3%	52.4%	NT	NT	20.0%	37.5%
Ceftazidime Clavulanic acid	45.9%	NT	0.0	NT	NT	NT	NT	NT	NT
Imipenem	57.4%	13%	72.7%	29.4%	57.1%	90.5%	NT	NT	NT
Meropenem	37.2%	13%	40.9%	29.4%	52.4%	23.8%	NT	NT	NT
Nitrofurantoin	49.7%	21.7%	13.6%	17.6%	61.9%	0.0	94.4%	70.0%	100.0%
Penicillin	NT	NT	NT	NT	NT	NT	33.3%	0.0%	12.5%
Cefoxitin	NT	NT	NT	NT	NT	NT	NT	15.0%	25.0%
Norfloxacin	4.4%	13.0%	13.6%	0.0%	23.8%	66.7%	33.3%	10.0%	37.5%
Ciprofloxacin	3.8%	21.7%	22.7%	5.9%	28.6%	71.4%	44.4%	10.0%	37.5%
Clindamycin	NT	NT	NT	NT	NT	NT	NT	55.0%	62.5%
Erythromycin	NT	NT	NT	NT	NT	NT	11.1%	5.0%	25.0%
H-gentamycin	NT	NT	NT	NT	NT	NT	66.7%	NT	NT
Netilmicin	NT	NT	NT	NT	NT	NT	NT	25.0%	75.0%
Novobiocin	NT	NT	NT	NT	NT	NT	NT	70.0%	87.5%
Tetracycline	NT	NT	NT	NT	NT	NT	38.9%	NT	NT
Doxycycline	NT	NT	NT	NT	NT	NT	NT	40.0%	50.0%
Teicoplanin	NT	NT	NT	NT	NT	NT	83.3%	NT	NT
Vancomycin	NT	NT	NT	NT	NT	NT	72.2%	100.0%	100.0%
Linezolid	NT	NT	NT	NT	NT	NT	88.9%	NT	NT
Chloramphenicol	NT	NT	NT	NT	NT	NT	83.3%	75.0%	87.5%

NT, not tested.

Nitrofurantoin, vancomycin, and chloramphenicol were particularly effective against gram-positive bacteria. Vancomycin, an antibiotic with restricted prescription, was found to inhibit 100% of *Staphylococcus* sp. and 72.2% of *Enterococcus* sp. Nitrofurantoin was also found to be effective against 94.4% of *Enterococcus* sp., 70.0% of *S. aureus* and 100% of *S. epidermidis.* Chloramphenicol, a rarely prescribed antibiotic, inhibited 83.3% of *Enterococcus* sp., 75.0% of *S. aureus*, and 87.5% of *S. epidermidis*. Ampicillin inhibited 66.7% of the *Enterococcus* sp. Furthermore, *Enterococcus* sp. showed an 88.9% susceptibility to linezolid and a 66.7% susceptibility to high concentration gentamycin.

## Discussion

The etiology, pathophysiology, and AS patterns of uropathogens have altered over time and place, which will continue to do so in the future (Ahmed et al., 2019). Identification of the organism and its AS is crucial for managing UTI. It exemplifies the importance of close collaboration and cooperation between the clinician and the microbiologist (Moue et al., 2015). This study aimed to assess the status of antimicrobial resistance among uropathogens and compare the situation in the Prayagraj region, the eastern part of North India. In our study, the prevalence of UTI was 79.9% since the inclusion criteria of patients was based on rigorous screening through a questionnaire [data not shown] by the clinicians. This prevalence rate is higher as compared to previous studies, which account for 45.7, 53.8, 65.4, and 37.3% in India, even though their inclusion criteria were symptom-based (Prakash and Saxena, 2013; Critchley et al., 2019; Patel et al., 2019; Sharma et al., 2020). The prevalence of UTI in our investigation correlates to a study conducted in the Mexican population, where 97.3% of patients excreted significant uropathogens and Ethiopia, where 90.1% of patients showed significant growth of uropathogens (García-Morúa et al., 2009; Seifu and Gebissa, 2018). According to several studies, the frequency of UTIs is higher in females than in males (Prakash and Saxena, 2013; Odoki et al., 2019; Malik et al., 2021). In concordance with previous research, our findings also indicate a higher prevalence of UTI in females (60.7%) than in males (39.3%). The proximity of the urethral meatus to the anus, the shorter urethra, sexual intercourse, incontinence, and improper toilet habits may contribute to a higher rate of UTI in females than in males (Prakash and Saxena, 2013). In our study, young females in the age of 18–50 years (reproductive age) showed a higher incidence of UTI, which is similar to the findings of the study in Meerut (26–36 years, 90.7%), Jaipur (21–50 years, 41.3%) and Ethiopia (20–29 years, 37.5%) as their anatomy makes them more vulnerable and prone to this disease (Sood and Gupta, 2012; Prakash and Saxena, 2013; Seifu and Gebissa, 2018). However, our study also revealed that elderly males (51–80 y) had a higher incidence of UTI (35.9%) than elderly females (25.7%). These findings mirrored studies conducted in Jaipur (Rajasthan), 47.3%; Meerut (Uttar Pradesh), 71.2%; Sonipat (Haryana), 58.3% India (Sood and Gupta, 2012; Prakash and Saxena, 2013; Malik et al., 2021). The leading causes of higher UTI incidence in elderly males might be attributed to the higher prevalence of benign prostate enlargement and neurogenic bladder (Lee and Kuo, 2017). Other researchers backed up similar findings, claiming that prostate disease in elderly males is responsible for the higher incidence of UTI (Rowe and Juthani-Mehta, 2013). The most common gram-negative bacteria isolated from samples in our investigation was *E. coli* (55.0%). These findings are consistent with those of several other published studies where the prevalence of *E. coli* was 97.0, 92.6, 74.0, 55.0, 49.3, 43.5, 41.9, and 40.0% (Arora et al., 2016; Odoki et al., 2019; Chen et al., 2020; Daoud et al., 2020; Ali et al., 2022; Huang et al., 2022; Jagadeesan et al., 2022; Komagamine et al., 2022). In our study, *Proteus* sp. (6.9%) and *K. pneumoniae* (6.6%) was the second and third most frequent bacteria reported, followed by *P. aeruginosa* (6.3%) and *Citrobacter* sp. (6.3%). *Proteus* sp. colonizes in the gastrointestinal tract of humans and causes UTI by ascending from the rectum to the urethral tissue and the urinary bladder. The increased prevalence of gram-negative bacteria from the *Enterobacteriaceae* family causing UTI can be attributed to several factors, including adherence to the uroepithelium due to urogenital mucosa colonization *via* adhesins, pili, fimbriae, and P-1 blood group phenotypic receptor (Terlizzi et al., 2017). *P. aeruginosa* is an unusual uropathogen that is primarily responsible for catheter-associated UTIs in adults. Its presence as the second commonest isolate (3/18, 16.7%) in the age group of 7–18 years needs further exploration. However, Bitsori et al. (2012) has suggested that with a history of previous UTI episodes, hospitalization, antibiotic use, malformations predisposing to UTIs, vesicourethral reflux, abnormal DMSA (dimercaptosuccinic acid) scan, longer hospitalization and surgery makes children more prone to *P. aeruginosa* UTI. The emergence of *Citrobacter* sp. as an uropathogen, especially in the age group >80 years, which is resistant to the majority of antibiotics, is alarming. *Citrobacter* sp. should no longer be ignored as commensal and proper surveillance in the antimicrobial sensitivity testing must be done (Sami et al., 2017).

In our study, 96.0% of the pathogens were MDR, compared to 91.3% in Nepal, 85.5% in Somaliland, 83.0% in Haryana, 45.1% in Tunisia, and 42.6% in China (Ben Ayed et al., 2019; Huang et al., 2022; Malik et al., 2021; Shilpakar et al., 2021; Ali et al., 2022). The inappropriate and indiscriminate use of broad-spectrum antibiotics and prolonged hospital stay are key etiological factors associated with MDR infections (Prestinaci et al., 2015). In our study, 40.4% of *E. coli* produced ESBLs, whereas other publications reported 25.2%; 35.7, 46.0, and 52–67% (Gharavi et al., 2021; Huang et al., 2022; Naushad et al., 2022; Sadeghi et al., 2022). ESBL producers hydrolyze and eliminate the majority of broad-spectrum beta-lactam antibiotics, increasing morbidity and mortality (Mahmud et al., 2020). Because ESBL-producing bacteria do not easily hydrolyze carbapenems, they are routinely used as first-line therapy in clinical settings. However, abuse of carbapenems, on the other hand, may make treatment of this type of bacterium more difficult (Gharavi et al., 2021). Antibiotic susceptibility revealed that amoxy-clav followed by ampicillin and cefepime were the most ineffective drugs against all identified gram-negative bacteria. In contrast, amikacin, gentamycin, and imipenem were the most susceptible drugs for gram-negative bacteria. These AS findings were consistent with prior research conducted in Sonipat (Haryana) and Meerut (UP) by other authors (Prakash and Saxena, 2013; Malik et al., 2021). In our study, tobramycin showed promising sensitivity to *P. aeruginosa*; however, according to a study conducted in Meerut, 60.0% of *P. aeruginosa* were resistant to tobramycin (Prakash and Saxena, 2013). In our study, imipenem and meropenem exhibited poor antimicrobial activity against gram-negative bacteria, in contrast to previous investigations in which carbapenem susceptibility was greater than 80.0% (Patel et al., 2019; Malik et al., 2021). Several studies have reported resistance to the β-lactam group of antibiotics, cephalosporins and fluoroquinolones, which is similar to that of our investigation, where a substantial decrease in sensitivity pattern was observed (Sood and Gupta, 2012; Sharma et al., 2020; Malik et al., 2021). Furthermore, in our study, nitrofurantoin exhibited significant susceptibility to *E. coli* but not to other *Enterobacteriaceae* (except *Citrobacter* sp.), which is consistent with a study conducted in Jaipur (Sood and Gupta, 2012). It is presumably due to irrational use of it in the past with insufficient dose and duration. Antibiotics showed considerably high sensitivity rates to gram-positive bacteria in our study, which was in concordance with the investigation conducted by other authors (Sood and Gupta, 2012; Patel et al., 2019).

## Conclusion

The main factor fueling AMR is improper usage of antibiotics that needs to be checked (Duan et al., 2021). According to the Infectious Diseases Society of America’s proposed regulations, empirical antibiotic treatment for UTI should be based on regional susceptibility data, drug accessibility, and patient history (Tamma et al., 2022). Resistance to bacterial uropathogens is becoming a public health issue in India. Many Indian cities and towns lack appropriate microbiological laboratories, leading to fewer microbiological assessments and increased empirical antibiotic use. Typically, urine samples are sent for microbiological testing only after treatment failure, recurrent or relapsing infection. Our findings emphasize the significance of local antibiotic resistance patterns, which may subsequently be used to develop hospital and regional antibiotic policies. To avoid/contain the emergence of antibiotic resistance in bacteria, the government must introduce laws requiring the prudent use of these antibiotics.

## Data availability statement

The original contributions presented in the study are included in the article/supplementary material, further inquiries can be directed to the corresponding authors.

## Ethics statement

The studies involving human participants were reviewed and approved by Institutional Ethical Committee for Human Research at Amity University Rajasthan, Jaipur, India (AUR/REG/2709). Written informed consent to participate in this study was provided by the participants’ legal guardian/next of kin.

## Author contributions

NJ, GN, and KB hypothesized and designed the research plan. AB and KB performed the data acquisition. KB and RK performed the experimental study. KB, NJ, AB, and GN did statistical analysis, interpretation of data, and manuscript preparation. GN, NJ, AB, and GA did final editing and reviewing. All the authors have reviewed the manuscript and approved the submitted version.

## References

[B1] Ahmed S. S. Shariq A. Alsalloom A. A. Babikir I. H. Alhomoud B. N. (2019). Uropathogens and their antimicrobial resistance patterns: Relationship with urinary tract infections. *Int. J. Health Sci.* 13 48–55. PMC6436442 30983946

[B2] Ali A. H. Reda D. Y. Ormago M. D. (2022). Prevalence and antimicrobial susceptibility pattern of urinary tract infection among pregnant women attending Hargeisa Group Hospital, Hargeisa, Somaliland. *Sci. Rep.* 12:1419. 10.1038/s41598-022-05452-z PMC8791963 35082366

[B3] Al-Zahrani J. Al Dossari K. Gabr A. H. Ahmed A. F. Al Shahrani S. A. Al-Ghamdi S. (2019). Antimicrobial resistance patterns of Uropathogens isolated from adult women with acute uncomplicated cystitis. *BMC Microbiol.* 19:237. 10.1186/s12866-019-1612-6 31666014 PMC6822473

[B4] Arora G. Kaur P. Agrawal D. (2016). Urinary tract infection in women of the rural population of Haryana: A rising problem. *Int. J. Reprod. Contracept. Obstet. Gynecol.* 5 4470–4474. 10.18203/2320-1770.ijrcog20164365

[B5] Ben Ayed H. Koubaa M. Hammami F. Marrakchi C. Rekik K. Ben Jemaa T. (2019). Performance of an Easy and Simple New Scoring Model in Predicting Multidrug-Resistant *Enterobacteriaceae* in Community-Acquired Urinary Tract Infections. *Open Forum Infect. Dis.* 6:ofz103. 10.1093/ofid/ofz103 PMC6441566 30949542

[B6] Bitsori M. Maraki S. Koukouraki S. Galanakis E. (2012). *Pseudomonas aeruginosa* urinary tract infection in children: Risk factors and outcomes. *J. Urol.* 187 260–264. 10.1016/j.juro.2011.09.035 22114821

[B7] Chen H. E. Tain Y. L. Kuo H. C. Hsu C. N. (2020). Trends in Antimicrobial Susceptibility of *Escherichia coli* Isolates in a Taiwanese Child Cohort with Urinary Tract Infections between 2004 and 2018. *Antibiotics* 9:501. 10.3390/antibiotics9080501 PMC7460002 32785113

[B8] Critchley I. A. Cotroneo N. Pucci M. J. Mendes R. (2019). The burden of antimicrobial resistance among urinary tract isolates of *Escherichia coli* in the United States in 2017. *PLoS One* 14:e0220265. 10.1371/journal.pone.0220265 31821338 PMC6903708

[B9] Daoud N. Hamdoun M. Hannachi H. Gharsallah C. Mallekh W. Bahri O. (2020). Antimicrobial Susceptibility Patterns of *Escherichia coli* among Tunisian Outpatients with Community-Acquired Urinary Tract Infection (2012-2018). *Curr. Urol.* 14 200–205. 10.1159/000499238 33488338 PMC7810217

[B10] Duan L. Liu C. Wang D. (2021). The General Population’s Inappropriate Behaviors and Misunderstanding of Antibiotic Use in China: A Systematic Review and Meta-Analysis. *Antibiotics* 10:497. 10.3390/antibiotics10050497 PMC8146421 33925971

[B11] Fazly Bazzaz B. S. Fork S. D. Ahmadi R. Khameneh B. (2021). Deep insights into urinary tract infections and effective natural remedies. *Afr. J. Urol.* 27 1–13.

[B12] Flores-Mireles A. L. Walker J. N. Caparon M. Hultgren S. J. (2015). Urinary tract infections: Epidemiology, mechanisms of infection and treatment options. *Nat. Rev. Microbiol.* 13 269–284. 10.1038/nrmicro3432 25853778 PMC4457377

[B13] García-Morúa A. Hernández-Torres A. Salazar-de-Hoyos J. L. Jaime-Dávila R. Gómez-Guerra L. S. (2009). Community-acquired urinary tract infection etiology and antibiotic resistance in a Mexican population group. *Rev. Mex. de Urol.* 69 45–48.

[B14] Gharavi M. J. Zarei J. Roshani-Asl P. Yazdanyar Z. Sharif M. Rashidi N. (2021). Comprehensive study of antimicrobial susceptibility pattern and extended spectrum beta-lactamase (ESBL) prevalence in bacteria isolated from urine samples. *Sci. Rep.* 11:578. 10.1038/s41598-020-79791-0 PMC7804094 33436687

[B15] Huang L. Huang C. Yan Y. Sun L. Li H. (2022). Urinary Tract Infection Etiological Profiles and Antibiotic Resistance Patterns Varied Among Different Age Categories: A Retrospective Study from a Tertiary General Hospital During a 12-Year Period. *Front. Microbiol.* 12:813145. 10.3389/fmicb.2021.813145 35154037 PMC8829000

[B16] Iacovelli V. Gaziev G. Topazio L. Bove P. Vespasiani G. Finazzi Agrò E. (2014). Nosocomial urinary tract infections: A review. *Urologia* 81 222–227. 10.5301/uro.5000092 25451882

[B17] Jagadeesan S. Tripathi B. K. Patel P. Muthathal S. (2022). Urinary tract infection and Diabetes Mellitus—Etio-clinical profile and antibiogram: A North Indian perspective. *J. Fam. Med. Prim. Care* 11 1902–1906. 10.4103/jfmpc.jfmpc_2017_21 PMC9254764 35800584

[B18] Kish L. (1965). *Survey Sampling*. (New York, NY: John Wiley & Sons, Inc).

[B19] Komagamine J. Yabuki T. Noritomi D. Okabe T. (2022). Prevalence of and factors associated with atypical presentation in bacteremic urinary tract infection. *Sci. Rep.* 12:5197. 10.1038/s41598-022-09222-9 PMC8956699 35338229

[B20] Kot B. (2019). Antibiotic Resistance Among Uropathogenic *Escherichia coli*. *Pol. J. Microbiol.* 68 403–415. 10.33073/pjm-2019-048 31880885 PMC7260639

[B21] Kucheria R. Dasgupta P. Sacks S. H. Khan M. S. Sheerin N. S. (2005). Urinary tract infections: New insights into a common problem. *Postgrad. Med. J.* 81 83–86. 10.1136/pgmj.2004.023036 15701738 PMC1743204

[B22] Lee C. L. Kuo H. C. (2017). Pathophysiology of benign prostate enlargement and lower urinary tract symptoms: Current concepts. *Ci Ji Yi Xue Za Zhi* 29 79–83. 28757771 10.4103/tcmj.tcmj_20_17 PMC5509197

[B23] Leung A. K. Wong A. H. Leung A. A. Hon K. L. (2019). Urinary tract infection in children. *Recent Pat. Inflamm. Allergy Drug Discov.* 13 2–18. 10.2174/1872213X13666181228154940 30592257 PMC6751349

[B24] Mahmud Z. H. Kabir M. H. Ali S. Moniruzzaman M. Imran K. M. Nafiz T. N. (2020). Extended-spectrum beta-lactamase-producing *Escherichia coli* in drinking water samples from a forcibly displaced, densely populated community setting in Bangladesh. *Front. Public Health* 8:228. 10.3389/fpubh.2020.00228 32626677 PMC7314906

[B25] Malik S. Rana J. S. Nehra K. (2021). Prevalence and antibiotic susceptibility pattern of uropathogenic *Escherichia coli* strains in Sonipat region of Haryana in India. *Biomed. Biotechnol. Res. J.* 5 80–87.

[B26] Mohapatra S. Panigrahy R. Tak V. Shwetha J. V. Sneha K. C. Chaudhuri S. (2022). Prevalence and resistance pattern of uropathogens from community settings of different regions: An experience from India. *Access. Microbiol.* 4:000321. 10.1099/acmi.0.000321 PMC8941965 35355869

[B27] Motbainor H. Bereded F. Mulu W. (2020). Multi-drug resistance of blood stream, urinary tract and surgical site nosocomial infections of *Acinetobacter baumannii* and *Pseudomonas aeruginosa* among patients hospitalized at Felegehiwot referral hospital, Northwest Ethiopia: A cross-sectional study. *BMC Infect. Dis.* 20:92. 10.1186/s12879-020-4811-8 32000693 PMC6993407

[B28] Moue A. Aktaruzzaman S. A. Ferdous N. Karim M. R. Khalil M. M. Das A. K. (2015). Prevalence of urinary tract infection in both outpatient department and inpatient department at a medical college setting of Bangladesh. *Int. J. Biosci.* 7 146–152. 10.12692/ijb/7.5.146-152

[B29] Mukherjee S. Mishra S. Tiwari S. (2020). Aetiological Profile and Antibiogram of Urinary Isolates Causing UTI in Patients Attending a Tertiary Care Hospital of Western Odisha. *J. Evol. Med. Dent. Sci.* 9 662–667. 10.14260/jemds/2020/144

[B30] Naushad V. A. Purayil N. K. Wilson G. J. Chandra P. Joseph P. Khalil Z. (2022). Epidemiology of urinary tract infection in adults caused by extended-spectrum beta-lactamase (ESBL)-producing *Enterobacteriaceae* - a case-control study from Qatar. *IJID Reg.* 3 278–286. 10.1016/j.ijregi.2022.05.001 35755476 PMC9216320

[B31] Odoki M. Almustapha A. A. Tibyangye J. Nyabayo M. J. Wampande E. Drago Kato C. (2019). Prevalence of Bacterial Urinary Tract Infections and Associated Factors among Patients Attending Hospitals in Bushenyi District, Uganda. *Int. J. Microbiol.* 2019:4246780. 10.1155/2019/4246780 PMC6397969 30906323

[B32] Olowe O. A. Ojo-Johnson B. B. Makanjuola O. B. Olowe R. A. Mabayoje V. O. (2015). Detection of bacteriuria among human immunodeficiency virus seropositive individuals in Osogbo, south-western Nigeria. *Eur. J. Microbiol. Immunol.* 5 126–130. 10.1556/EuJMI-D-14-00036 25883800 PMC4397854

[B33] Patel H. B. Soni S. T. Bhagyalaxmi A. Patel N. M. (2019). Causative agents of urinary tract infections and their antimicrobial susceptibility patterns at a referral center in Western India: An audit to help clinicians prevent antibiotic misuse. *J. Fam. Med. Prim. Care* 8 154–159. 10.4103/jfmpc.jfmpc_203_18 PMC6396617 30911498

[B34] Prakash D. Saxena R. S. (2013). Distribution and antimicrobial susceptibility pattern of bacterial pathogens causing urinary tract infection in urban community of Meerut city, India. *ISRN Microbiol.* 2013:749629. 10.1155/2013/749629 PMC3830820 24288649

[B35] Prestinaci F. Pezzotti P. Pantosti A. (2015). Antimicrobial resistance: A global multifaceted phenomenon. *Pathog. Glob. Health* 109 309–318. 10.1179/2047773215Y.0000000030 26343252 PMC4768623

[B36] Revelas A. (2012). Healthcare - associated infections: A public health problem. *Niger. Med. J.* 53 59–64. 10.4103/0300-1652.103543 23271847 PMC3530249

[B37] Rowe T. A. Juthani-Mehta M. (2013). Urinary tract infection in older adults. *Aging Health* 9 519–528 10.2217/ahe.13.38 24391677 PMC3878051

[B38] Sadeghi M. Ebrahim-Saraie H. S. Mojtahedi A. (2022). Prevalence of ESBL and AmpC genes in E. coli isolates from urinary tract infections in the north of Iran. *New Microbes New Infect.* 45:100947. 10.1016/j.nmni.2021.100947 PMC8693013 34984104

[B39] Sami H. Sultan A. Rizvi M. Khan F. Ahmad S. Shukla I. (2017). Citrobacter as a uropathogen, its prevalence and antibiotics susceptibility pattern. CHRISMED. *J. Health Res.* 4 23–26. 10.4103/2348-3334.196037

[B40] Seifu W. D. Gebissa A. D. (2018). Prevalence and antibiotic susceptibility of Uropathogens from cases of urinary tract infections (UTI) in Shashemene referral hospital, Ethiopia. *BMC Infect. Dis.* 18:30. 10.1186/s12879-017-2911-x 29320984 PMC5763535

[B41] Sharma P. Netam A. K. Singh R. (2020). Prevalence and in vitro antibiotic susceptibility pattern of bacterial strains isolated from tribal women suffering from urinary tract infections in District Anuppur, Madhya Pradesh, India. *Biomed. Res. Ther.* 7 3944–3953. 10.15419/bmrat.v7i8.625

[B42] Shilpakar A. Ansari M. Rai K. R. Rai G. Rai S. K. (2021). Prevalence of multidrug-resistant and extended-spectrum beta-lactamase producing Gram-negative isolates from clinical samples in a tertiary care hospital of Nepal. *Trop. Med. Health* 49:23. 10.1186/s41182-021-00313-3 PMC7948344 33691795

[B43] Sood S. Gupta R. (2012). Antibiotic resistance pattern of community acquired uropathogens at a tertiary care hospital in Jaipur, Rajasthan. *Indian J. Community Med.* 37 39–44. 10.4103/0970-0218.94023 22529539 PMC3326806

[B44] Tamma P. D. Aitken S. L. Bonomo R. A. Mathers A. J. van Duin D. Clancy C. J. (2022). Infectious diseases society of america guidance on the treatment of AmpC β-Lactamase-producing Enterobacterales, Carbapenem-resistant *Acinetobacter baumannii*, and *Stenotrophomonas maltophilia* infections. *Clin. Infect. Dis*. 74, 2089–2114. 34864936 10.1093/cid/ciab1013

[B45] Terlizzi M. E. Gribaudo G. Maffei M. E. (2017). UroPathogenic *Escherichia coli* (UPEC) Infections: Virulence Factors, Bladder Responses, Antibiotic, and Non-antibiotic Antimicrobial Strategies. *Front. Microbiol.* 8:1566. 10.3389/fmicb.2017.01566 28861072 PMC5559502

